# Nonhuman primate models of pediatric viral diseases

**DOI:** 10.3389/fcimb.2024.1493885

**Published:** 2024-12-03

**Authors:** Vidya Vijayan K. K., Kristina De Paris

**Affiliations:** ^1^ Department of Microbiology and Immunology, University of North Carolina, Chapel Hill, NC, United States; ^2^ Center for AIDS Research, University of North Carolina, Chapel Hill, NC, United States; ^3^ Children’s Research Institute, University of North Carolina, Chapel Hill, NC, United States

**Keywords:** pediatric disease, nonhuman primate models, viral pathogens, infant immunity, virus-host interactions

## Abstract

Infectious diseases are the leading cause of death in infants and children under 5 years of age. *In utero* exposure to viruses can lead to spontaneous abortion, preterm birth, congenital abnormalities or other developmental defects, often resulting in lifelong health sequalae. The underlying biological mechanisms are difficult to study in humans due to ethical concerns and limited sample access. Nonhuman primates (NHP) are closely related to humans, and pregnancy and immune ontogeny in infants are very similar to humans. Therefore, NHP are a highly relevant model for understanding fetal and postnatal virus-host interactions and to define immune mechanisms associated with increased morbidity and mortality in infants. We will discuss NHP models of viruses causing congenital infections, respiratory diseases in early life, and HIV. Cytomegalovirus (CMV) remains the most common cause of congenital defects worldwide. Measles is a vaccine-preventable disease, yet measles cases are resurging. Zika is an example of an emerging arbovirus with devastating consequences for the developing fetus and the surviving infant. Among the respiratory viruses, we will discuss influenza and Severe Acute Respiratory Syndrome Coronavirus 2 (SARS-CoV-2). We will finish with HIV as an example of a lifelong infection without a cure or vaccine. The review will highlight (i) the impact of viral infections on fetal and infant immune development, (ii) how differences in infant and adult immune responses to infection alter disease outcome, and emphasize the invaluable contribution of pediatric NHP infection models to the design of effective treatment and prevention strategies, including vaccines, for human infants.

## Introduction

Globally, infectious diseases remain a significant cause of morbidity and mortality in the pediatric population (Frenkel, 2021; Perin et al., 2022; Kelly et al., 2024). Despite substantial progress in reducing child death in recent decades, infectious diseases cause approximately 25% of deaths in the neonatal period and up to one-third of deaths in children under 5 years (Bhutta and Black, 2013; Semmes et al., 2020; Perin et al., 2022). The neonatal period of the first 28 days of life represents the most vulnerable period, with about 17 deaths per 1,000 live births in 2022 (UNICEF, 2024). The majority of neonatal deaths are due to preterm birth complications or other intrapartum events, with relatively fewer infectious causes (Perin et al., 2022). Congenital infection, depending on the pathogen, maternal pathogen-specific immunity, and trimester of infection, however, can have detrimental consequences for the fetus ranging from death to lifelong health sequalae (Megli and Coyne, 2022; De Rose et al., 2023). Death in children under the age of 5 years can be primarily attributed to infectious diseases, leading causes being diarrhea, upper respiratory tract infections, malaria, measles, tuberculosis, and HIV (Perin et al., 2022). The burden of neonatal and infant morbidity and mortality is highest in low- and middle-income countries, where it is associated with high health care, economic and societal costs.

Since the start of the 20^th^ century, the global community has been facing high-impact and fast-spreading outbreaks of new or neglected pathogens that are difficult to manage. In the decade between 2010 and 2020 alone, more than 1,400 epidemic events were reported (Board, 2023). The vast majority of outbreaks (e.g., Middle East Respiratory Syndrome, Ebola, avian influenza, Zika) were caused by viral pathogens. Therefore, this review will focus on pediatric infections of viral origin.

Considering ethical concerns and limitations in sample access and sample size for studies of viral infections in human infants, relevant pediatric animal models that optimally reflect host-pathogen interactions and disease progression are required. Such animal models should allow infection with the human virus or an equivalent animal species-specific virus, replication of the virus, clinical symptoms reflecting human disease, and induction of cellular and humoral immune responses (Ruiz et al., 2013). Small animal models, particularly mouse models, provide the advantage of relatively inexpensive housing and care, rapid reproduction with multiple offsprings, and vast resources, including genetic modifications, to study pathogenesis and host immunity (Estes et al., 2018; Lemaitre et al., 2021). However, the phylogenetic distance from humans and differences in early immune development limit the utility of these models for pediatric infections.

In contrast, the close phylogenetic relationship of NHPs with humans renders them highly relevant as model systems for human diseases (Baxter and Griffin, 2016; Estes et al., 2018). Moreover, many, although not all, human viruses can infect certain NHP species directly or require minimal adaptation (Estes et al., 2018). Among the NHP, Great Apes, Old World monkeys, and New World monkeys have been utilized to understand pathogenesis and develop prevention and treatment strategies for various diseases. The Great Apes, including chimpanzees, bonobos, gorillas, and orangutans share ~98% genetic similarity with humans. Chimpanzees, gorillas and orangutans of have been used previously as animal model for infection with human immune deficiency virus (HIV) (Fultz et al., 1989; Nath et al., 2000), hepatitis B virus (HBV) (Wieland, 2015) and hepatitis C virus (HCV) (Lanford et al., 2001). Due to ethical and conservational concerns, invasive biomedical research with these species is now banned in the USA, Europe, and many other countries. Most NHP studies are conducted directly at or in close collaboration with National Primate Research Centers (NPRCs). The USA has a network of seven federally funded NPRCs with the overarching goal to improve human health through biomedical research involving NHP. The work of NPRCs is overseen and regulated by the Office of Laboratory Animal Welfare at the National Institutes of Health. Studies have to adhere to the International Guiding Principles of Biomedical Research Involving Animals, be aligned with the Guide for the Care and Use of Laboratory Animals developed by the Committee on Care and Use of Laboratory Animals of the Institute of Laboratory Resources at the National Resource Council (2011) and be conducted in accordance with the American Association for Accreditation of Laboratory Animal Care Standards. The Animal Plant and Health Inspection Service (APHIS) of the US Department of Agriculture is responsible for enforcing the US Animal Welfare Act. The application of human principles to the welfare of research animals was proposed in 1959 and is best known as the Three Rs, replacement, reduction and refinement of animals in biomedical research (Russell and Burch, 1959), guiding principles for NHP research.

In fact, overall, NHPs represent a very small proportion (<1%) of animals used in biomedical research (National Academy of Sciences, Engineering, and Medicine, 2023). Old World monkeys represent the most commonly NHP models harnessed in biomedical research, although New World monkeys, in particular marmosets, have gained more acceptance as reliable models for human diseases in recent years (Herron et al., 2024). Macaques, including rhesus (*Macaca mulatta*), cynomolgus (*M. fascicularis*), and pig-tailed (*M. nemestrina*) macaques, but also vervet monkeys, have been employed as model systems for viral diseases. Macaques share approximately 94% of their genetic code with humans, are highly similar in anatomy, physiology, neurophysiology and neurodevelopment, the reproductive system, and, especially relevant for the purpose of this review, in immune system development (Estes et al., 2018).

Pediatric NHP models offer the opportunity for highly relevant translational studies of virus-host interactions that can inform diagnostics, prevention, intervention and treatment strategies tailored to the immune system of the vulnerable population of human infants on a global scale. This review will briefly discuss the unique factors of the early life immune system, highlight the similarities between humans and NHPs, and present representative pediatric NHP models of viral infections.

## The lifespan of non-human primates compared to humans

**Figure 1:**
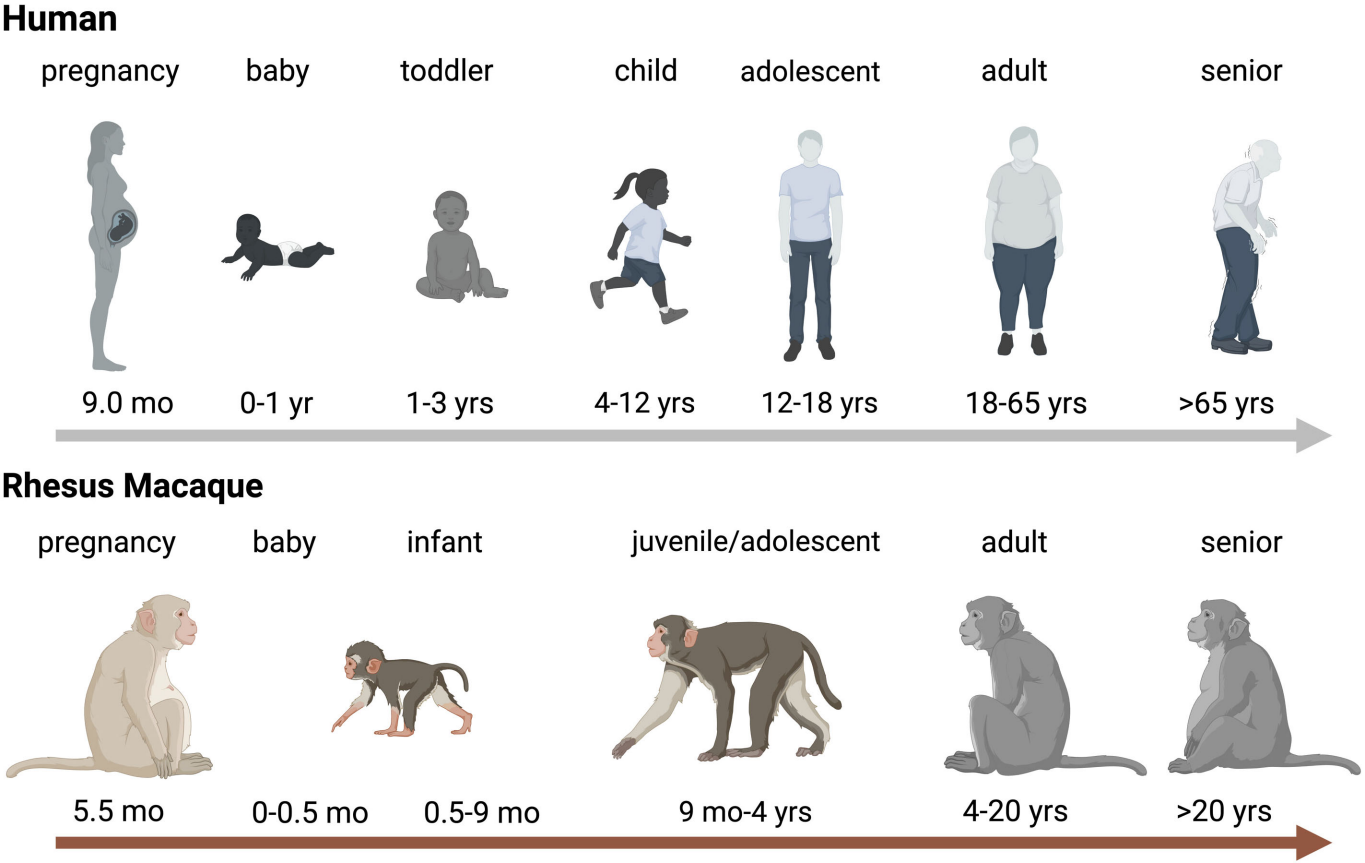
Age Comparison between Humans and Rhesus Macaques. Schematic representration of aging in humans (top panel) and rhesus macaques (bottom). Critical age periods are indicated by name on top of the human or rhesus macaque icon and time spans are indicated below the relevant icons. Abbreviations stand for: wk=week, mo=months, and yrs=years. The figure was created in BioRender (De Paris, 2024a).

It is important to first define how NHP age translates to human age. There are no clear guidelines, but age ranges for infant, juvenile, and adult NHPs are highly comparable between US National Primate Research Centers (NPRCs) and align well with the US Primate Aging Database (Kemnitz, 2019) and The macaque Website by the United Kingdom’s National Centre for the Replacement, Refinement and Reduction of Animals in Research (National Centre for the Replacement RaRoAiR, 2023). Differences in age ranges are partly due to the specific factors applied to assess aging. A study determining the impact of thimerosal-containing pediatric vaccines in rhesus macaques, evaluated toxicity by neuro-behavioral developmental milestones assuming that rhesus macaques age 4 times faster that human infants (Boothe et al., 1985; Curtis et al., 2015). Another study estimated that aging occurs approximately 3 times faster compared to humans based on aging-associated pathology changes and comorbidities in humans (Simmons, 2016). For the purpose of this review, based on our findings related to developmental changes in immune function of infant rhesus macaques (Van Rompay et al., 2004; Oxford et al., 2015; Jensen et al., 2016b), we will define the following age groups. The neonatal period in rhesus macaques comprises ≤7 days compared to 28 days in humans. Human infants can be divided into babies, toddlers, pre-school and school age children, categories that are more difficult to define in rhesus macaques. At 2 weeks of age, a baby rhesus macaque is more similar to a human toddler and infancy extends up to the time of weaning, that, in dam-reared infants, can be as early as 3 months and as late as 9 months. The juvenile age starts post weaning and adolescence and sexual maturity are reached between 3 and 4 years of age, with females generally maturing earlier than their male counterparts (**Figure 1**). Sexually mature animals are considered young adults between 4-6 years, and adult macaques can get more >20 years old. Therefore, the aging process in rhesus macaques (and other NHPs) is compressed compared to humans.

## Non-human primates reflect human early life immune system development

To develop and utilize relevant pediatric NHP models of common, newly emerging, or-remerging viral infections in human infants, it is important that NHP immune ontogeny is equivalent to human immune development. Several studies, as outlined below, have provided evidence that pregnancy initiation, placenta formation, as well as fetal and postnatal immune system development are comparable between humans and Old World monkeys, especially rhesus macaques (DeMaria et al., 2000; Golos, 2004; Bakkour et al., 2014; Tarantal et al., 2022).

### Placentation and fetal development

The placenta is regarded as the gateway to connecting mother and fetus, the interface that allows the transportation of nutrients, immunity, and several other factors, such as oxygen from mother to fetus. Therefore, it is important to understand placentation in NHPs as it relates to humans. Following fertilization, the human embryo undergoes interstitial implantation with massive decidual development. Placentation in NHPs is characterized by somewhat superficial implantation due to a less developed decidua lobe (Roberts et al., 2012). However, trophoblast invasion of the uterine wall is analogous to human (Furukawa et al., 2014) and both human and NHP embryo-maternal tissue apposition and exchange are highly comparable (Hendrickx et al., 2000). A recent transcriptomic analysis of human and macaque placenta revealed that the majority of human placental genes were shared between the two species (Rosenkrantz et al., 2021). Furthermore, comparative studies of human and macaque embryos demonstrated overall morphological similarities in fetal development. Thus, although shorter in duration, the fetal growth period and organogenesis follow a similar trajectory in humans and NHPs (Hendrickx et al., 2000).

### Immune ontogeny

The immune milieu at the maternal-fetal interface and during the perinatal period is increasingly recognized as being critical in shaping lifelong immunity and susceptibility to diseases (Jain, 2020). The development of the human immune system begins as early as three weeks after conception and continues after birth and into childhood (Simon et al., 2015). The intrauterine development of the immune system is a feature common to both humans and NHPs (Makori et al., 2003). Batchelder and colleagues reported similar temporal and anatomical sequences in immune ontogeny in both species (Batchelder et al., 2014). During fetal development, the primary site of hematopoiesis is the fetal liver, with subsequent hematopoiesis being established in bone marrow. Comparable to humans, innate immune cells are detected earlier in gestational age compared to T and B cells, the main adaptive immune cell types (Seddiki and Le Grand, 2021), and the ability of the immune system to distinguish self from non-self antigens is acquired prior to birth (Gonzalez et al., 2011).

Postnatally, the sudden onslaught of pathogens and foreign entities, combined with the establishment of host microbiota, renders the largely naive newborn and infant immune system highly susceptible to infections. The distinctive immunological profile involving cell intrinsic hypo-responsiveness concomitant with enhanced activation of immunosuppressive mechanisms and antigen-inexperienced adaptive functions explain the unique vulnerability of infants to infection (Prendergast et al., 2012). Yet, mounting evidence suggests that the early infant immune systems is a highly regulated functional and dynamic network of competent molecular and cellular components - rather than an immature immune scheme – that follows a specific trajectory and that immune development follows a specific trajectory (Olin et al., 2018; Yu et al., 2018; Lee et al., 2019). We and others have demonstrated that, despite accelerated aging of rhesus macaques compared to human infants, key steps of the functional maturation of innate and adaptive responses are analogous (DeMaria et al., 2000; Pitcher et al., 2002; Van Rompay et al., 2005a; Abel et al., 2006; Nguyen et al., 2011; Van Rompay and Jayashankar, 2012; Oxford et al., 2015; Boesch et al., 2016; Jensen et al., 2016b; Jensen et al., 2016a; Boesch et al., 2017; Tolbert et al., 2019; Crowley et al., 2021). Potential differences in antibody responses related to Fc receptor function and dominance of Ig subclasses, however, need to be considered, especially when evaluating novel vaccine candidates (Boesch et al., 2016).

### Impact of microbiome on immune development

The composition of the infant’s microbiota is an important regulator of the activation and the development of the pediatric immune system and the infant’s resistance or susceptibility to various infections and diseases in later life (Gensollen et al., 2016; Yao et al., 2021). Gestational age at birth, delivery mode, antibiotic use, feeding practice, weaning time, and environment all impact the infant’s microbiome establishment (Nunez et al., 2021). The microbiota and their metabolites provide signals that prime and activate innate immune cells in early life, and this activation is critical for the functional maturation of innate effector cells to protect against infectious pathogens (Sanidad and Zeng, 2020; Dogra et al., 2021). The colonization of microbes at mucosal surfaces is essential for the maturation of mucosal IgA responses (Cerutti et al., 2011; Gustafson et al., 2014).

The macaque microbiota, in particular the gastrointestinal, shares several dominant, bacterial taxa with the human microbiota, but differences exist at the species level (Chen et al., 2018; Brenchley and Ortiz, 2021). Host genetics likely account for differences in microbiota between these two primate species, but the environment, including captivity, feeding practices, and food variety of NHPs in biomedical research, are additional factors that determine microbiome composition (Brenchley and Ortiz, 2021). Nonetheless, there are important commonalities in the establishment of the host microbiota after birth. The microbiome composition, similar to humans, is distinct in formula versus breast-fed infant macaques (Ardeshir et al., 2014). Furthermore, these differences in microbiota are associated with distinct immune profiles that persist throughout infancy (Narayan et al., 2015).

### Maternal antibody transfer

As the immune system in early life transitions from a functionally naïve to an antigen-experienced entity, transplacentally transferred maternal antibodies represent a key defense mechanism against many infectious diseases. However, only circulating maternal IgG can be transferred transplacentally from the mother to the fetus (Langel et al., 2022). Multiple studies have documented the correlation between placental function and the magnitude of passively transferred antibodies with the early protection against viral diseases (Albrecht et al., 1977; Fouda et al., 2018; Pou et al., 2019). For example, RSV-specific maternal IgG has been associated with protection from RSV infection until 6 months of age (Albrecht et al., 1977). Post birth, various cellular and soluble immune factors, including all classes of immunoglobulins, can be passed from the mother to the infant by breastfeeding.

Great apes and Old World monkeys express neonatal Fc receptors on their placental membrane cells and, analogous to humans, utilize the same mechanism to transplacentally transfer antibodies to the fetus for early protection against disease (Pentsuk and van der Laan, 2009). Despite few studies of maternal antibody transfer in NHPs, findings revealed that, as observed in human pregnancy, only IgG can cross the placenta and antibody transfer is highest in the last trimester (Shen et al., 2014; Sartoretti and Eberhardt, 2021). Recently, it has been proposed that NHP require a second receptor to allow transfer of IgG antibodies from the placenta to the fetus (Rosenberg et al., 2023). There are also some differences in the levels of antibodies that can be transferred across the placenta (Sartoretti and Eberhardt, 2021). Furthermore, the antibody half-life in NHPs has been estimated to be approximately 8.3 days compared to the half-life of antibodies in humans, which ranges from 10 to 25 days (Citron et al., 2021).

Thus, despite some differences (e.g., allelic polymorphisms, functional hierarchy of antibody subclasses), the general developmental path of the immune system in NHPs is comparable to that in humans, supporting the translational value of NHP models for pediatric viral infections.

## Pediatric NHP models of viral infections

**Figure 2:**
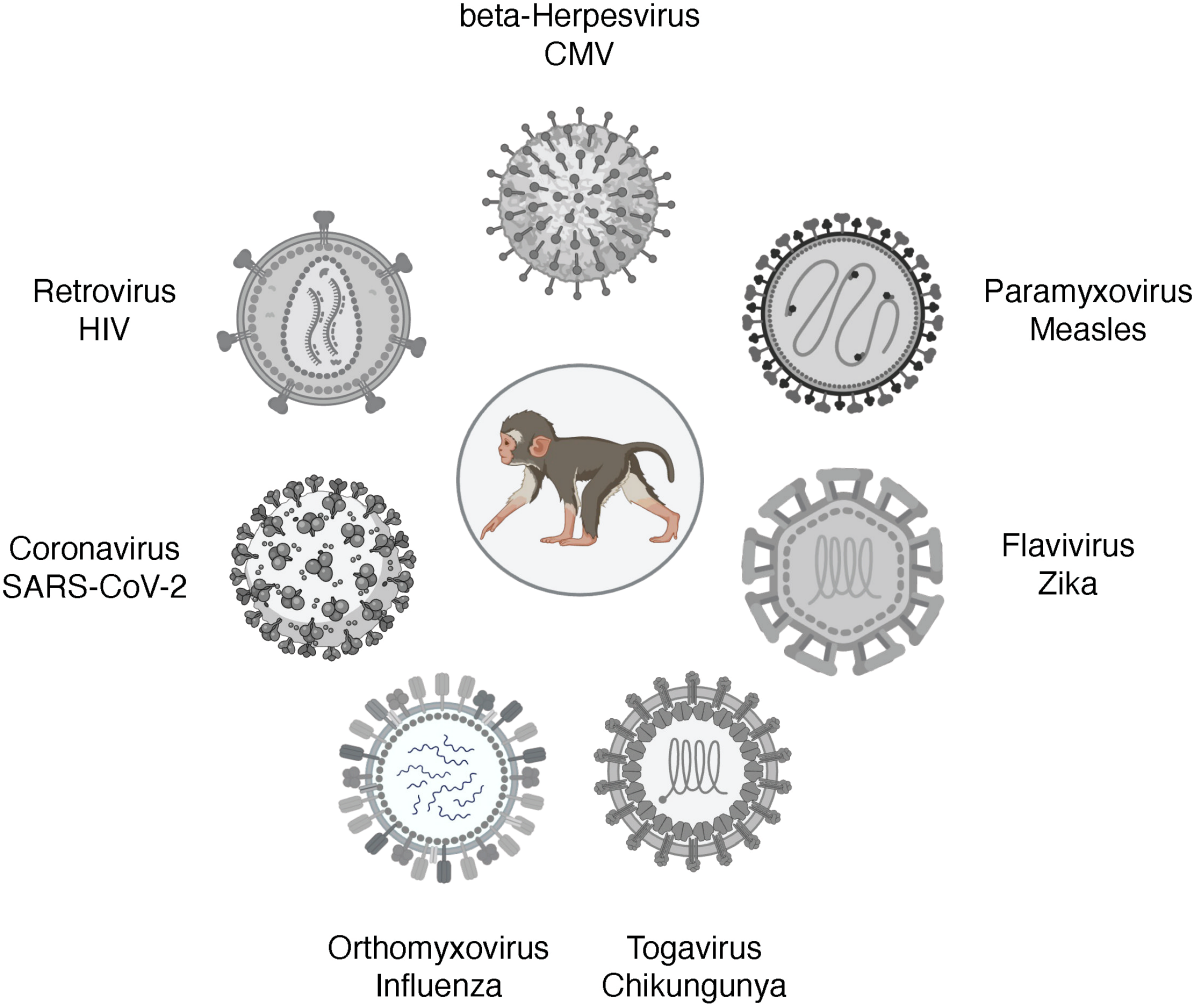
Pediatric infection models of viral diseases in NHP. Figure created in BioRender.

An excellent review of NHP models of viral diseases in adults has been recently published (Estes et al., 2018). This review will discuss NHP models of viruses that can cause congenital infections, respiratory diseases in early life, and HIV (**Figure 2**). Cytomegalovirus (CMV) remains the most common cause of congenital defects worldwide. Measles is a vaccine-preventable disease, yet measles cases are resurging. Zika is an example of an emerging arbovirus with devastating consequences for the developing fetus and the surviving infant. Among the respiratory viruses, we will discuss influenza models and pediatric NHP models of SARS-CoV-2. We will finish with HIV as an example of a lifelong infection without a cure or vaccine.

## Cytomegalovirus

Human Cytomegalovirus (HCMV) is a double-stranded DNA beta herpes virus from the family *Herpesviridae*. Seroprevalence of HCMV in adults is estimated to range from 50% in high-income countries to up to 90% or more in LMICs (Britt and Alford, 1996; Zuhair et al., 2019; Griffiths and Reeves, 2021). In LMICs, CMV is acquired in early childhood (Zuhair et al., 2019). Although CMV infection in healthy individuals is generally asymptomatic, CMV will persist lifelong and can be reactivated. Furthermore, despite strong and persistent antibody and T cell responses to HCMV (Sylwester et al., 2005), re-infection with HCMV does occur (Yamamoto et al., 2010). In immunocompromised individuals (e.g., transplant or cancer patients, people living with HIV), HCMV infection is a serious health concern. Moreover, HCMV infection remains the most common congenital infection worldwide, with about one in 150 newborns being affected (Kenneson and Cannon, 2007; Lanzieri et al., 2014). Thus, CMV infection is associated with major health care burdens and costs.

The species-specificity of herpesviruses is a challenge for the development of relevant animal models. Yet, chimpanzee CMV (cCMV) and rhesus CMV (RhCMV) are genetically closely related to HCMV, and the rhesus macaque model of RhCMV infection has been extensively utilized to study transmission, pathogenesis, persistence, host immunity, CMV immune evasion, and vaccine strategies (Barry et al., 2006; Itell et al., 2017a; Hill, 2018). Captive rhesus macaques in National Primate Research Centers (NPRC) in the USA acquire RhCMV early in life, with 100% seroprevalence at the time of sexual maturity (Vogel et al., 1994). To study RhCMV pathogenesis under more controlled conditions, the NPRCs in the USA have also established specific pathogen-free (SPF), including RhCMV-free, breeding colonies (Barry and Strelow, 2008).

HCMV is primarily shed in saliva and urine, and oral HCMV transmission is considered the main route of horizontal transmission, and this holds true for natural RhCMV infection as well (Kaur et al., 2018). Oral RhCMV infection models have been developed for all age groups (Lockridge et al., 1999; dela Pena et al., 2012; Yue et al., 2022). Infection was established with natural isolates of RhCMV (dela Pena et al., 2012), the wildtype (wt)-like full-length RhCMV strains UCD52 and UCD59 (Yue et al., 2022), and with RHMCV 68-1 (Lockridge et al., 1999). The RhCMV strains 68-1, as well as RHCMV 180.92, have been propagated in fibroblasts and it was later determined that both strains contain numerous mutations (Lilja and Shenk, 2008; Oxford et al., 2011; Yue et al., 2016). In particular, RhCMV 68-1, but not RhCMV 180.92, lacks the surface pentameric complex (PC; UL128-131) that is important for epithelial and endothelial cell tropism and presents a major target for neutralizing antibodies in the host (Rivailler et al., 2006; Lilja and Shenk, 2008; Oxford et al., 2011; Yue et al., 2016; Itell et al., 2017b). Therefore, while both RhCMV 68-1 and RhCMV 180.92 can be used to study horizontal transmission, RhCMV68-1 is not suitable for transplacental transmission studies of RhCMV if dams that are infected by the oral exposure (Rivailler et al., 2006; Assaf et al., 2014; Bialas et al., 2015).

Congenital HCMV infection can occur in both seronegative and seropositive pregnant women (Fowler et al., 2003). The earlier in pregnancy the mother acquires CMV, the more severe the outcome is for the baby. The spectrum of teratogenic effects includes microcephaly, ventriculomegaly, growth restrictions, neurocognitive impairments, vision and hearing loss (Kirschen and Burd, 2023). In fact, CMV infection accounts for most cases of hearing loss in infants in the US (Morton and Nance, 2006; Ross KBF, 2008). However, as the rate of intrauterine transmission in seropositive women is very low, it is likely similarly low in rhesus macaques. In earlier studies, when pregnant dams were infected with RhCMV by the intrauterine route, RhCMV infection caused abortions, intrauterine growth restrictions, neurological defects, and/or hearing loss (Tarantal et al., 1998; Chang et al., 2002; Itell et al., 2017b), reflecting the severe complications associated with congenital HCMV infection. In contrast, also similar to human HCMV pathogenesis, a recent study documented that preexisting RhCMV immunity in pregnant dams reduces placental RhCMV transmission when the dam is reinfected in the first or early second trimester and fetal loss is prevented (Mostrom et al., 2023). In the latter study, seropositive rhesus macaques in their late first or early second trimester of pregnancy were CD4 T cell depleted and then infected with UCD52 and a full-length recombinant RhCMV expressing SIVgag (FL-RhCMVΔRh13.1/SIVgag) or with RhCMV 180.92. Reinfection was confirmed by increased viral shedding in urine and saliva. Virus, however, could not be amplified by PCR from amniotic fluids of RhCMV-positive reinfected dams, whereas virus was detectable in primary RhCMV-infected dams (Mostrom et al., 2023). It should be noted though that neither the magnitude of viremia nor of plasma binding antibodies in response to primary acute RhCMV infection predict whether vertical transmission will occur in otherwise immunocompetent dams (Otero et al., 2023). Thus, while pre-existing antibodies in seropositive dams can prevent vertical transmission, antibodies induced to primary RhCMV infection might be induced too late to prevent transplacental transfer. Depending on the trimester when experimental RhCMV infection is introduced, the congenital RhCMV infection model can be utilized to test for early diagnostic markers of congenital infection, type and severity of developmental defects, persistence of symptoms during childhood, and to assess the efficacy of intervention and treatment strategies. There is a need for a more detailed analysis of innate and adaptive immune responses that can protect against vertical transmission *in utero* (Itell et al., 2017c). Moreover, assessment of immune parameters should include quantitative as well as functional, qualitative readouts. NHP models of breastmilk transmission of RhCMV are lacking. Considering that other herpesviruses, such as Kaposi’s Sarcoma Virus, can also be transmitted via breastfeeding, such a model would be of relevance to study pediatric HCMV and KSHV infections in low-income countries of the sub-Saharan African region where prevalence is high and HIV coinfection might further impact pathogenesis outcome.

Despite extensive research (Abel et al., 2008; Abel et al., 2011), a CMV vaccine is not yet available. Most CMV vaccine strategies have focused immunogen design on glycoprotein B (gB) or PC. The HCMV and RhCMV gB and PC proteins share approximately 60% amino acid homology and exert comparable functions (Kravitz et al., 1997; Anderholm et al., 2016). In humans and rhesus macaques, infection with CMV induces high magnitude T cell responses that are maintained throughout the lifetime, as well as persistent antibody responses. Yet, these responses cannot prevent infection with a different strain of CMV, emphasizing the challenge of designing an effective vaccine that can protect against CMV infection. Furthermore, at least in rabbits, a multivalent CMV gB mRNA vaccine was not able to increase the breadth of the antibody response (Wang et al., 2024). In the case of vertical CMV transmission, the PC appears to be required for villous, but not for extravillous trophoblasts (Wussow et al., 2017; Naing et al., 2020), implying that alternative vaccine targets should be explored to achieve protection against horizontal and vertical transmission (Deere et al., 2019; Li et al., 2022). Furthermore, and as pointed out above, considering the ubiquitous nature of CMV in humans, vaccine studies in rhesus macaques should be conducted in seropositive animals to account for potential interference by preexisting immunity (Webster et al., 2021). The inclusion of pregnant dams in NHP vaccine testing studies to prevent vertical transmission of CMV provides a unique opportunity in the development of prevention strategies against congenital CMV infection.

## Measles virus

Measles virus (MeV), an enveloped single-stranded negative-sense RNA virus belonging to the Morbillivirus genus of the Paramyxoviridae family, is the causative agent of measles. Measles is highly contagious and transmitted by inhalation and spread by circulating lymphoid cells in the body. In immunocompetent hosts, measles represents an acute viral infection associated with a maculopapular skin rash, fever, and often also respiratory symptoms. Yet, in infants and young children, measles may result in more severe morbidity and fatal outcome (Moss and Griffin, 2012). Despite the introduction of a safe and effective live attenuated measles vaccine in 1963 that is part of the WHO Extended Program of Immunization in infants, globally over 100,000 children succumb to death from MeV infection annually (Minta et al., 2023). The MeV vaccine is only administered to infants 9 months and older (Sanchez-Schmitz and Levy, 2011; Griffin, 2018) due to vaccine interference by maternal antibodies (Albrecht et al., 1977). Even in the absence of maternal antibodies though, responses are less robust in younger than older infants and two doses of the vaccine are needed to achieve MeV-specific antibody response in >95% of vaccinated infants (Nair et al., 2007). Thus, there remains a window of susceptibility during which maternal antibodies wane and vaccine-induced seroconversion rates are low, leaving infants susceptible to infection (Sanchez-Schmitz and Levy, 2011). Another reason why measles cases have been resurging in some countries is a 1998 report that falsely claimed that the measles vaccine is contributing to developmental disorders and autism (Montgomery and Wakefield, 1997). Although the data in the manuscript were refuted by numerous studies, measles vaccination rates dropped, and the original message of Wakefield’s manuscript (Montgomery and Wakefield, 1997) continues to fuel worries by parents about vaccine safety.

Experimental MeV infection of monkeys has been reported over a hundred years ago (Blake and Trask, 1921a; Blake and Trask, 1921b; Blake and Trask, 1921c). Pediatric rhesus and cynomolgus macaques provide an excellent NHP model for the study of measles pathogenesis and vaccine-induced protective immunity since they reproduce most of the pathologic and immunological aspects of measles in infants, including rash and fever (Hall et al., 1971; van Binnendijk et al., 1994; McChesney et al., 1997; Zhu et al., 1997; de Swart et al., 2007; de Swart, 2009). Experimental infection is possible using human MeV strains, although some models have passaged human clinical MeV isolates in macaques prior to use in this NHP model. There are a total of 24 genotypes of MeV that are based on the sequence diversity of the MeV hemagglutinin (H) and nucleocapsid (N) genes (Bankamp et al., 2024). Genotype A is now extinct but gave rise to the first MeV vaccine strain, the Edmonston strain, that was isolated from an infected child (Rota et al., 2011). Wildtype measles virus, such as Edmonston and Bilthoven best recapitulate human measles disease in rhesus macaques (Griffin, 2018). However, infections of juvenile rhesus or cynomolgus macaques with different MeV strains in parallel revealed that extended *in vitro* passage of MeV strains in non-primate cells markedly reduces disease symptoms in NHP models and that rhesus macaques are more susceptible to MeV disease compared to cynomolgus macaques (van Binnendijk et al., 1994; Auwaerter et al., 1999; Valsamakis et al., 1999).

Use of fluorescently-labeled MeV in rhesus macaques demonstrated that lymphoid cells, and in particular B cells, are highly susceptible to virus infection (McChesney et al., 1997; de Swart et al., 2007) and that one of the entry receptors for MeV is CD150, also called signaling lymphocyte activation molecule (SLAM). The use of MeV strains engineered with enhanced green fluorescent protein (EGFP) were also instrumental in defining the steps leading to skin rash development after MeV infection. The analysis of tissue sections from intratracheally or aerosol MeV-infected cynomolgus and rhesus macaques demonstrated that migrating MeV-positive lymphocytes infect CD150-positive skin resident lymphoid and myeloid cells in the dermis, from where the infection spreads to nectin-4-positive keratinocytes in the basal and then the superficial epidermis (Laksono et al., 2021). Eventually, immune cells will be recruited and eliminate these MeV-infected cells causing hyperemia and edema that are apparent as the typical morbilliform skin rash (Laksono et al., 2021). These findings in NHP were confirmed using ex vivo human skin sheets, highlighting how studies in NHP models can inform human MeV pathogenesis.

The pediatric NHP model of MeV infection was and remains critical for MeV vaccine development (Enders et al., 1960; Katz et al., 1960). Numerous strategies have been attempted to develop alternative vaccines that would allow the administration of the measles vaccine to newborns or infants younger than 9 months (Osterhaus et al., 1994). Towards this goal, a MeV DNA vaccine tested in infant rhesus macaques was shown to be effective in reducing MeV viremia in the presence of passively administered MeV-specific antibody (Premenko-Lanier et al., 2003; Premenko-Lanier et al., 2004). Other studies have explored subunit vaccines that target the hemagglutinin (H) or fusion (F) proteins that are crucial in MeV binding to its receptors, CD46, CD150, or nectin-4 [reviewed in (Griffin et al., 2008)]. Recently, a fusion inhibitory peptide that was administered through a nebulizer could protect juvenile cynomolgus macaques against intratracheal MeV infection (Reynard et al., 2022). In another study, using a squirrel monkey model, a small molecule polymerase inhibitor, ERDRP-0519, was tested for its antiviral efficacy when given prophylactically at 12 hours prior to MeV infection or therapeutically at day 3 or day 7 post intranasal infection with a primary MeV strain circulating in the human population (Wittwer et al., 2021). While the development of severe and generalized rash was prevented in all animals, only the squirrel monkey receiving the polymerase inhibitor prophylactically or by day 3 post infection exhibited reduced viremia compared to control animals (Wittwer et al., 2021). Pediatric NHP models also provide the opportunity to conduct extensive safety assessment of vaccines. Thimerosal, an organomercury compound with anti-septic and anti-fungal activity, has been included in some pediatric vaccines, such as earlier MeV, some influenza vaccines, and the Hep B vaccine. As mercury is neurotoxic (Chang, 1977), infant rhesus macaques were vaccinated according to the human pediatric vaccine schedule with the measles, mumps, rubella (MMR) vaccine, the Hep B, Diphteria/Tetanus/acellular Pertussis (DTaP)/and Haemophilus influenza type B (HiB) vaccines, or saline as control (Boothe et al., 1985; Curtis et al., 2015). Assuming a 4:1 age progression in NHP compared to humans, study animals were evaluated longitudinally for age-relevant developmental milestones applying a battery of comprehensive neurobehavioral tests. The results of the former study did not provide evidence of any neurodevelopmental toxicity of thimerosal-containing pediatric vaccines (Curtis et al., 2015). These findings confirmed an earlier study conducted by the US Food and Drug Administration assessing the safety of thimerosal-containing vaccines (Ball et al., 2001). Nonetheless, it was recommended to remove thimerosal from vaccines administered to children under the age of 6.

Combined, the findings from these studies demonstrate important contributions of NHP models to the understanding of virus transmission, virus entry, target cells and tissues of virus replication, and disease pathogenesis (Kempe and Fulginiti, 1965), but many open questions remain. Potential research topics could focus on (i) the type of MeV virulence factors that need to be attenuated to achieve vaccine protection, (ii) the specific correlates of vaccine-mediated protection, (iii) mechanisms of maternal antibody interference, and (iv) novel vaccine platforms that allow measles vaccination early after birth.

## Zika virus

Zika virus (ZIKV) is a mosquito-born, single-stranded RNA virus belonging to the *Flaviviridae* family. Until the outbreak in 2015, ZIKV infection was mainly confined to Africa, with occasional outbreaks in Asia. The emergence of ZIKV in South and Central America was associated with serious complications in pregnant women and their infants. ZIKV infection during pregnancy resulted in severe teratogenic effects including microcephaly, intracranial calcification, and even fetal death (Steele, 2016; Bhagat et al., 2021). The congenital malformations following ZIKV infection are now recognized as congenital Zika syndrome (CZS). CZS comprises a spectrum of phenotypes ranging from congenital microcephaly to more subtle ocular and auditory abnormalities, cognitive motor deficits, and seizure (Haese et al., 2021). Importantly, the severity of congenital ZIKV infection results in major developmental impairments with lifelong consequences.

NHP present a host in the sylvatic life cycle of the ZIKV (Vorou, 2016). As NHP have a similar placental organization and the development of the fetal organ, nervous, and immune system is highly comparable to humans, NHP, especially rhesus and cynomolgus macaques, have emerged as a highly relevant model to study ZIKV infection. In contrast, neurogenesis and brain development differ significantly between rodents and humans (Miner et al., 2016; Zhang et al., 2023) and mouse models of ZIKV infection further require the introduction of deletions in the interferon signaling pathway to allow for efficient virus replication (Zhang et al., 2023). Congenital ZIKV NHP models, however, have recapitulated many of the features that are present in human congenital ZIKV infection, including fetal demise, placental villous damage, periventricular brain lesions, and fetal retinal dysplasia (Dudley et al., 2016; Mohr, 2018). These studies primarily used the Puerto Rico Zika strain PRVABC59, with infection by the subcutaneous route to mimic exposure via mosquito bites (Dudley et al., 2016; Mohr, 2018). Importantly, parallel studies at different NPRCs with other ZIKV strains isolated in Brazil, Cambodia, and France, resulted in similar outcomes, validating the rhesus macaque model. The NHP studies also demonstrated that the timing of infection in pregnancy is a key determinant for the severity of the disease outcome; ZIKV is about twice as likely to infect the placenta early in pregnancy compared to later in gestational age (Brasil et al., 2016; Hirsch et al., 2018; Martinot et al., 2018). The inflammatory response to ZIKV infection was shown to result in placental villi damage and decreased oxygen supply to the fetus (Hirsch et al., 2018). Histopathological evaluation of fetal and neonatal brains of ZIKV-infected pigtail and rhesus macaque revealed lesions in occipital-parietal lobes, abnormal white matter, and impaired neuronal development (Adams Waldorf et al., 2016; Morrison and Diamond, 2017; Coffey et al., 2018; Martinot et al., 2018), abnormalities reflecting those observed in human fetuses with ZIKV infection [reviewed in (Haese et al., 2021)]. Thus, the studies of congenital ZIKV infection in the NHP model have expanded the understanding of congenital ZIKV infection and provide the opportunity to examine direct damage in the brain, an organ that cannot be sampled in living humans. In fact, in 2016, the FDA Medical Countermeasures Initiative had specifically partnered with NPRCs, in particular the California NPRC, to define ZIKV distribution and persistence in multiple tissues, as study that would not be possible to conduct in humans (US Food and Drug Administration, 2016).

Geographically, ZIKV infection overlaps with Dengue virus (DENV) infection, another arthropod-borne member of the *Flaviviridae* family. As some of the DENV and ZIKV antigens are structurally similar, it is important to consider preexisting DENV immunity on ZIKV infection and vertical ZIKV transmission (Gaspar-Castillo et al., 2023). Indeed, studies in both rhesus and cynomolgus macaques demonstrated more severe CZS outcomes in dams with preexisting Dengue immunity (Ausderau et al., 2021; Crooks et al., 2021; Saron et al., 2023). These findings further emphasize the high translational value of these NHP models for ZIKV research to develop novel prevention strategies. Potential ZIKV vaccine candidates have been evaluated in NHP models (Abbink et al., 2016; Dowd et al., 2016; Pardi et al., 2017; Gaudinski et al., 2018). More studies are needed though to define immune correlates of protection, the timing of vaccination in pregnant women in relation to ZIKV infection, and in the presence or absence of DENV immunity.

With the exception of genetic testing for medical reasons, it is unethical to collect placental and fetal tissue samples during pregnancy or infant brain samples post birth. Thus, studies of transplacental virus transfer and mechanistic pathways leading to teratogenic complications are not possible in humans. Furthermore, long-term longitudinal studies of human infants who were born with CZS or milder ZIKV infection symptoms are needed to identify long-term health consequences and late-onset deficits. Relevant animal models of ZIKV transmission and infection could provide mechanistic insights into ZIKV pathogenesis, guide antenatal care, and inform treatment strategies in humans to ameliorate and improve health outcomes in affected children as they become adolescents and adults. It is encouraging that studies have been initiated to test promising drugs aimed at attenuating neurological deficits of ZIKV infection in infant macaques (Medina et al., 2023).

## Chikungunya virus

Chikungunya virus (CHIKV) is a single-stranded RNA virus belonging to the arthropod-borne alphaviruses in the *Togaviridae* family. Originally identified in Africa (Schwartz and Albert, 2010), CHIKV has now spread worldwide and become endemic (ECDC, 2024). Worldwide travel and climate change are two of the main reasons why arboviruses were and are able to expand their geographical distribution and rapidly spread worldwide (Azevedo Rdo et al., 2015; Organization, 2017). So far, in 2024 alone, it is estimated that approximately 450,000 people contracted CHIKV and about 160 people died of CHIKV infection. The majority of cases occur in the Americas, followed by Asia, with rare cases reported in Africa and Europe (ECDC, 2024).

CHIKV infection in humans is of rapid onset and manifests itself by high fever, headaches, rash, and polyarthralgia (Ganesan et al., 2017). While acute CHIKV infection resolves quickly within 5 to 7 days (Schwartz and Albert, 2010), polyarthralgia can last from weeks to months to even years (Mavalankar et al., 2008; Wahid et al., 2017; Suhrbier, 2019). Morbidity and mortality are highest in the elderly, neonates, and adults with comorbidities (Rao et al., 2008; Economopoulou et al., 2009; de Souza et al., 2024). In one of the major CHIKV outbreaks, the La Réunion outbreak in 2005, the case fatality rate was estimated to be 1 in 1,000 (Mavalankar et al., 2008). During the La Réunion CHIKV outbreak, vertical transmission of CHIKV was first reported (Gerardin et al., 2008). Although vertical transmission of CHIKV is relatively rare, it can result in intrauterine death or preterm delivery (Couderc and Lecuit, 2009; Ferreira et al., 2022). Neonatal CHIKV infection can result in sepsis, encephalitis, and neurodevelopmental delays (Gerardin et al., 2014; Ferreira et al., 2022; Faria et al., 2024). Thus, the long-term consequences of CHIKV infections that are acquired by horizontal or vertical transmission are associated with major health care and societal burdens. Antiviral drugs and vaccines specifically targeting CHIKV are not available. Progress for prevention and intervention strategies is hampered in part by the lack of an animal model that fully recapitulates the spectrum of symptoms that can be induced by CHIKV infection in humans.

Macaques represent the most common NHP Model for CHIKV infections, and both rhesus and cynomolgous macaques serve as natural hosts for CHIKV in the sylvatic cycle (Broeckel et al., 2015; Haese et al., 2021). Importantly, the persistence of CHIKV that had been observed in human patients could be recapitulated in several tissues of rhesus macaques, even when viremia was cleared after CHIKV infection (Labadie et al., 2010). CHIKV infected macaques also presented with fever, sometimes rash, leukopenia, and musculoskeletal symptoms, such as joint swelling (Broeckel et al., 2015; Haese et al., 2021). Furthermore, aged rhesus macaques (>17 years) presented with higher viremia and more severe disease than adult animals at age 6 to 13 years (Messaoudi et al., 2013). The NHP model of CHIKV infection has been successfully applied to study virus dissemination, early innate responses, target cells, viral reservoir, host immunity, and to test vaccines and therapeutics (Akahata et al., 2010; Chen et al., 2010; Labadie et al., 2010; Kam et al., 2014; Pal et al., 2014; Roy et al., 2014; Broeckel et al., 2015; Roques et al., 2017; Haese et al., 2021; Beddingfield et al., 2022). The efficacy of the live-attenuated vaccine VLA1553, that is based on the La Reunion strain but shows cross-neutralization of other circulating Asian strains, was tested by transferring sera from human adults immunized in the phase I trial of the VLA1553 vaccine to cynomolgus macaques one day prior to challenge with the WT CHIKV LR2006-OPY1 (Roques et al., 2017; Roques et al., 2022). Human sera were pooled at distinct timepoints post vaccination to determine what neutralizing antibody titer was required to mediate protection. A micro-plaque neutralization test (μPRNT50) titer >150 was effective in protection cynomolgus macaques against plasma viremia (Roques et al., 2022). Importantly, this titer was concordant with the PRNT_80_ titer previously determined with sera from a different human study (Yoon et al., 2020). A phase 3 clinical trial has since confirmed the immunogenicity and safety of the single-shot Chikungunya VLA1553 vaccine in healthy adults (Schneider et al., 2023; McMahon et al., 2024).

Vertical transmission studies have been performed in rhesus macaques and demonstrated, consistent with human findings, that maternal infection during the prepartum period is not associated with infection of the fetus (Chen et al., 2010). We are not aware of NHP studies testing peripartum or intrapartum CHIKV infection that would then also allow the study of CHIKV outcomes in neonatal and infant macaques. This lack of pediatric CHIKV infection models represents a clear gap, especially as the numbers of CHIKV infection increase and new lineages emerge as a result of the global spread (Barr and Vaidhyanathan, 2019).

## Influenza virus

The influenza virus is a common viral respiratory tract pathogen that belongs to the *Orthomyxoviridae* family with a segmented negative-strand RNA genome (Pleschka, 2013). The virus causes epithelial necrosis and inflammation that can potentially result in pneumonia (Taubenberger and Morens, 2008). Young infants and the elderly have a higher risk for more severe disease, secondary complications, and mortality (Macdonald and Bortolussi, 2009; Langer et al., 2023). In the USA, seasonal influenza vaccines are approved for infants age 6 months or older, leaving the most vulnerable infants at risk for influenza infection. Moreover, the available data show the rate of seroconversion against H1N1 strains in young infants is only 29–32% following two doses of the inactivated trivalent influenza vaccine (Halasa et al., 2008). Most human influenza outbreaks are caused by influenza A (IAV) or influenza B (IBV) viruses (Pleschka, 2013). Influenza subtypes are determined by the viral surface proteins hemagglutinin (HA) and neuraminidase (NA). Influenza viruses undergo antigenic drift and shift to evade host immunity, with “drift” referring to the gradual accumulation of mutations in HA or NA, while antigenic shift refers to the emergence of novel influenza viruses that were previously not known to infect humans. These new influenza subtypes can emerge because segmented genomes of co-circulating influenza viruses can be reassorted and influenza viruses can cross species barriers (O’Donnell and Subbarao, 2011). In the absence of pre-existing immunity, these novel subtypes can cause a pandemic. Examples of these new subtypes include the H3N2 viruses that emerged in the late 1960^ties^, the 2009 H1N1 swine influenza virus outbreak in humans, and the so called “bird flu viruses”, mainly of the H5N1 subtype of avian origin, that have been circulating in the human population since 2003 (Taubenberger and Morens, 2010). Many questions regarding the role of gene reassortment, species crossing, and pathogenic potential for pandemic outbreaks remain unanswered. Relevant animal models of influenza infection could provide mechanistic insights, increase our ability to predict pandemics, and inform vaccine design.

The similarity in lung development and physiology between humans and NHP (Tran et al., 2004; Bouvier and Lowen, 2010; Miller et al., 2017) implies high relevance of NHP models for influenza studies. Furthermore, examination of wild NHP in their habitats confirmed evidence of natural infection of NHP by influenza viruses (Karlsson et al., 2012). Influenza viruses, including seasonal (e.g., H1N1), pandemic (e.g., H3N2), and emerging (e.g., H5N1) viruses, can infect both Old and New World monkeys (O’Donnell and Subbarao, 2011). Yet, the host receptor for HA, sialic acid (SA), expresses different galactose linkages in distinct species and on different cell types of the upper and lower respiratory tract, and these differences in virus-target cell interaction and the SA receptor distribution in the upper and lower respiratory tract can significantly impact disease outcome (O’Donnell and Subbarao, 2011).

Indeed, early studies of influenza infection in NHP had reported that, in contrast to rodent models of IAV infection, intranasal (IN) inoculation of NHP with IAV did not result in disease, whereas intratracheal (IT) inoculation with influenza virus resulted in virus shedding, leukopenia, fever, nasal discharge, and nonproductive cough (Marois et al., 1971; Berendt, 1974; Bodewes et al., 2010), symptoms typical for human IAV infection. These distinct outcomes in pathogenesis were thought to be due to differences in SA receptor expression at the site of virus exposure (O’Donnell and Subbarao, 2011). Later studies confirmed that infection of only the upper, but not the lower, respiratory tract after exposure of cynomolgus macaques to H3N1 or H5N1 could be attributed to tissue-specific patterns of SA expression in humans and NHP (Kuiken et al., 2003b; Rimmelzwaan et al., 2003; van Riel et al., 2007). In contrast, infection of cynomolgus macaques by multiple routes, including IT, per-oral (tonsillar; PO), and intra-ocular (IO), to H5N1 resulted in severe disease (Rimmelzwaan et al., 2001). Infection of cynomolgus macaques, seronegative for circulating H1N1 and H3N2 reference viruses, with the pandemic 1918 virus that killed an estimated 50 million people by combined IT, IN, PO, and IO inoculation resulted in severe respiratory disease requiring euthanasia (Kobasa et al., 2007). The same multi-route infection model with the 1918 virus conducted approximately 15 years later in rhesus macaques yielded very different results, only very mild symptoms were observed (Chan et al., 2022). This finding implied that rhesus compared to cynomolgus macaques are a less suitable model to study IAV infection. The same investigators then decided to repeat the experiment in cynomolgus macaques, and although they observed more severe clinical symptoms compared to those in rhesus macaques, none of the cynomolgus macaques succumbed to infection or met the clinical criteria for euthanasia (Chan et al., 2022) that were described in the earlier study (Kobasa et al., 2007). The studies were not only conducted more than a decade apart, but also at different primate research facilities that had access to NHP of different origin. Furthermore, the cynomolgus macaques in the earlier study were between 9 and 19 years of age, whereas most animals in the recent study were young adults (Chan et al., 2022). Thus, both differences in the genetic background and in age of the cynomolgus macaques could have impacted disease outcome. In humans, it is well established that people aged 65 years and older experience more severe disease and mortality after influenza infection than young adults (Langer et al., 2023). Age as risk factor for disease severity is consistent with the results of a study comparing influenza outcome in adult and aged rhesus macaques infected with the 2009 H1N1 pandemic virus (swine flu) (Josset et al., 2012).

Rhesus macaques may also not be optimal to study pediatric influenza infection. Only a single study could be identified that tested IAV infection in infant rhesus macaques (Clay et al., 2014), and in that study, animals were between 6 to 11 months of age, corresponding to human infants aged 18 months to 4 years, an age group eligible for influenza vaccination. Furthermore, despite infant rhesus macaques being inoculated by the intranasal and intratracheal route, viral RNA in tracheal samples were very low, indicative of replication occurring primarily in the upper respiratory tract (Clay et al., 2014). However, *in vitro* H1N1 infection of infant versus adult airway epithelial cell cultures of rhesus macaques showed higher virus replication in infant compared to adult airway epithelial cells and that increased virus replication was associated with reduced type I interferon responses in infant airway epithelial cells (Clay et al., 2014). A direct comparison of PR8 infection (also by combined IT and IN route) in neonatal (6 to 11 days of age) versus adult (> 3 years) African Green Monkeys (AGMs) clearly demonstrated higher virus replication in infant compared to adult tracheal AGM samples (Holbrook et al., 2015). Furthermore, infants had reduced influenza-specific IgG levels in the respiratory tract and influenza-specific IgA was lower in both the respiratory tract and plasma of infant compared to adult AGMs (Holbrook et al., 2015). Similarly, influenza-specific neutralizing IgG antibody titers were lower in tracheal samples of infant compared to adult AGMs, whereas systemic neutralizing responses were comparable in both age groups (Holbrook et al., 2015). These findings are consistent with delayed mucosal responses in human infants (see above). In fact, the investigators provided evidence that bronchus-associated lymphoid tissue (BALT) was less developed in the infant versus adult AGMs (Holbrook et al., 2015). In contrast, regulatory T cells of tracheobronchial lymph nodes were increased in infant compared to adult AGMs. In a subsequent study, the group by Alexander-Miller further characterized the antibody response to HA (Clemens et al., 2020). They demonstrated that the hierarchy of the five main epitopes in the HA head differed for plasma IgM between infant and adult AGMs at day 8 but was similar in both age groups by day 14. At day 14 post infection, there was no difference in systemic IgG epitope hierarchy (Cb dominance), whereas IgA antibodies were directed predominantly against the Sb epitope in infants, whereas both Sb- and Cb-specific IgA dominated the adult response. Interestingly, infant and adult AGM also mounted plasma IgG responses against the HA stem, and these responses were of similar magnitude and avidity in infant and adult AGMs. HA stem-specific IgG was also detectable in bronchoalveolar fluids of both age groups, although at much lower concentrations. HA stem-specific IgA, however, was only detected in the plasma of 1 adult AGM and in BAL of 2 adult AGMs, whereas induction of HA stem-specific IgA was not detectable in infant AGMs (Clemens et al., 2020). This finding is important as some universal influenza vaccine strategies are exploring HA stem immunogens. To overcome the more immunoregulatory milieu in infants and to enhance antibody response, the same group also utilized the pediatric AGM model to test different adjuvants for their ability to enhance infant antibody response to influenza vaccines. Indeed, immunization of neonatal AGMs with flagellin-adjuvanted inactivated PR8 vaccine compared to nonactive flagellin-adjuvanted IPR8 vaccine was able to significantly increase systemic IgG responses, antibodies had higher affinity and persisted for 6 months (Holbrook et al., 2016). In a separate study, they tested the TLR7,8 agonist R848 and found that R848-adjuvanted IPR8 enhances the antibody responses even more (Clemens et al., 2021). Furthermore, neonatal vaccination of adjuvanted inactivated PR8 vaccine was able to elicit HA stem-specific antibody responses after the second immunization, and these responses were more sustained in infant AGMs that had received the R848-adjuvanted vaccine compared to the flagellin vaccine (Clemens et al., 2022). The studies also revealed that influenza vaccines adjuvanted with R848 induced higher antibody responses and improved neutralizing function in female compared to male infant AGMs (Holbrook et al., 2024). The studies in the pediatric AGM influenza model highlight how a model system can be effectively used to understand pathogenesis and to develop and test various vaccine strategies to protect against IAV infection, and how vaccine design can be optimized to enhance efficacy in the pediatric population.

## Severe acute respiratory syndrome Coronavirus 2

Coronaviruses with pathogenic potential, in particular severe respiratory disease, in humans include the Middle East Respiratory Syndrome coronavirus (MERS-CoV), SARS-CoV-1 and SARS-CoV-2 (Docea et al., 2020). All three viruses are enveloped single-stranded RNA viruses belonging to the *Coronaviridae* family. The viruses are transmitted through infectious aerosols and droplets. SARS-CoV-2, an enveloped single-strand RNA virus, is the causative agent of the global COVID-19 pandemic (Zhou et al., 2020) that resulted in millions of deaths worldwide. Some individuals present with long-lasting symptoms, now referred to as long-COVID, after primary infection (Davis et al., 2023). Similar to influenza, SARS-CoV-2 proved especially fatal in the elderly population, but unlike influenza, SARS-CoV-2 presented as largely mild disease in infants and children (She et al., 2020). However, a subset of children developed atypical skin manifestations and severe inflammation of several different organs, referred to as multisystem inflammatory syndrome in children (MIS-C) (Akca et al., 2020; Malcangi et al., 2022). The highly contagious nature of SARS-CoV-2, the rapid global spread, and the high mortality and morbidity of the virus caused severe healthcare challenges and an almost economic standstill. At the same time, the global pandemic promoted a collaborative effort in vaccine development that culminated in the fast-track of emergency approvals for vaccines to protect against SARS-CoV-2 infection (Chavda et al., 2022).

Since the fatal outbreak of MERS, NHP, especially rhesus and cynomolgus macaques, had been developed as model systems to study coronavirus-induced transmission, pathogenesis, prevention and intervention strategies (Kuiken et al., 2003a; van Doremalen and Munster, 2015; Munster et al., 2020; Williamson et al., 2020; Lemaitre et al., 2021; McMahan et al., 2021; Van Rompay et al., 2021). Although the majority of children exhibited reduced severity of clinical symptoms compared to adults infected with SARS-CoV-2, the enormous global burden and extent of COVID-19 associated morbidity and mortality justified the simultaneous testing of pediatric SAR-CoV-2 vaccines. Indeed, while it normally takes years to license vaccines for all age groups, the scale of the SARS-CoV-2 pandemic and its impact on every area of life from healthcare to education, travel and economy, required an expedited start of clinical vaccine trials with adolescents and children, and these were indeed initiated within a year of adult clinical trials (Polack et al., 2020; Baden et al., 2021; El Sahly et al., 2021; Gilbert et al., 2021; Anderson et al., 2022; Creech et al., 2022; Munoz et al., 2023). Our group tested two different vaccine platforms in approximately two-month old infant rhesus macaques, comparing the immunogenicity and efficacy of a two-dose adjuvanted SARS-CoV-2 spike protein regimen and a preclinical version of the Moderna SARS-CoV-2 mRNA vaccine (Garrido et al., 2021; Milligan et al., 2023). Both vaccines induced rapid antibody responses, including neutralizing antibodies, to the vaccine immunogen. Although antibody titers declined over time, neutralizing antibodies persisted for one year. SARS-CoV-2 specific T cell responses appeared to increase over time and were also detectable up to one year post immunization. Both antibody and T cell responses were cross-reactive to newly emerging variants of SARS-CoV-2. Although the adjuvanted protein vaccine had induced consistently higher neutralizing antibody titers compared to the mRNA vaccine, animals in both vaccine groups were protected against severe lung pathology and exhibited significantly reduced viral burden in the lower respiratory tract after challenge with delta SARS-CoV-2 virus, a virus heterologous to the wildtype SARS-CoV-2 spike protein of the vaccine, at one year after immunization (Milligan et al., 2023). Overall, the adjuvanted protein vaccine more efficiently and rapidly controlled virus replication in the upper respiratory tract compared to the mRNA vaccine. Protection in infant rhesus macaques vaccinated with the adjuvanted protein vaccine was primarily driven by neutralizing antibodies, whereas both neutralizing antibodies and T cell responses contributed to protection in mRNA-vaccinated rhesus macaques. The data were important as this study used a 3-fold lower dose (30 mcg) of the Moderna vaccine compared to the vaccine dose for human adults (100 mcg) that had received emergency approval. Furthermore, our results indicated that infant rhesus macaques, despite receiving a lower vaccine dose, developed antibody responses comparable in magnitude to those observed in human adults (Garrido et al., 2021), and that these antibody responses persisted for at least 12 months, consistent with vaccine-induced B cell memory (Milligan et al., 2023). At the same time, the findings implied that vaccine efficacy can be optimized and, if possible and available, multiple distinct vaccine platforms should be evaluated in parallel. In contrast to the MERS and SARS-CoV-1 pandemic, SARS-CoV-2 has evolved and appears to have become endemic. Therefore, there is a need to not only develop seasonal influenza, but also SARS-CoV-2 vaccines and, as outlined above, pediatric NHP models may help in testing and advancing novel vaccines into human infant clinical trials.

Studies of SARS-CoV-2 infection in rhesus macaque dam-infant pairs contributed to the identification of age-dependent differences in pathogenesis outcome after SARS-CoV-2 infection. Although small in sample size, two studies reported similar findings (Fovet et al., 2022; Langel et al., 2022). Despite no differences in viral load between dams and their neonates, infants mounted a stronger type I interferon response compared to their mother, while the immune response in dams was biased towards inflammation (e.g., induction of IL-6) and hypoxia (Fovet et al., 2022; Langel et al., 2022). Furthermore, the induction of neutralizing antibodies appeared to be faster in infants compared to their dams. Thus, infants mount a faster antiviral response to SARS-CoV-2 infection compared to adults that might translate into more efficient control of viral burden and less tissue pathology. Interestingly, a high IL-6 response and low type I interferon responses were also observed in adult cynomolgus macaques infected with the pandemic 1918 influenza strain that resulted in severe disease (Kobasa et al., 2007). Why adult macaques respond with a stronger inflammatory response that ultimately leads to more tissue damage and pathology remains to be determined.

## Human immunodeficiency virus

HIV is a lentivirus that belongs to the family of *Retroviridae*. Although HIV emerged globally in the early 1980^ties^ resulting in the Acquired Immunodeficiency Syndrome (AIDS) pandemic, the virus had jumped from NHP to humans many years earlier in Africa (reviewed in (Sharp and Hahn, 2011). Since its emergence, approximately 40 million lives have been lost to HIV and it is estimated that about an equal number of people are living with HIV (WHO, 2024). Clinical and basic research of HIV transmission and pathogenesis have transformed the outcome of HIV infection from a fatal disease to a manageable chronic disease with normal life expectancy. HIV is primarily transmitted sexually but can also be acquired via contaminated blood sources by percutaneous exposure, and through vertical transmission. There are several outstanding reviews of adult NHP HIV infection models (e.g (Del Prete et al., 2016; Van Rompay KKA. Tackling, 2017; Veazey and Lackner, 2017; Estes et al., 2018).,). Here, building on earlier reviews (Jayaraman and Haigwood, 2006; Marthas and Miller, 2007; Abel, 2009; Van Rompay and Jayashankar, 2012), the focus will be on pediatric HIV infection models in NHP and how they contribute to clinical management of pediatric HIV.

Vertical transmission of HIV can occur *in utero*, peripartum and by breastfeeding. Intrauterine infection models of simian immunodeficiency virus (SIV), a virus closely related to HIV, were developed in rhesus and pigtail macaques (Davison-Fairburn et al., 1990; McClure et al., 1991; Fazely et al., 1993; Ochs et al., 1993). Dr. Amedee established a rhesus macaque model to study breastmilk transmission of HIV that assessed transmission frequency, viral evolution and host immune responses (Amedee et al., 2003; Amedee et al., 2004; Rychert et al., 2006). However, as HIV or SIV acquisition by breastfeeding is a rare event in human or rhesus macaque infants, respectively, it was cost-prohibitive to continue further development of this model.

Infection of newborn or young infant macaques with SIV by the intravenous route (Van Rompay et al., 1992) were utilized early in the pandemic to test early antiviral drug regimens. (e.g., AZT, Zidovudine) approved for human adults with HIV for their potential use in the pediatric population (Van Rompay et al., 1992; Van Rompay et al., 1995). These initial studies in NHP paved the way for the initiation of human clinical trials to test the efficacy of Zidovudine in women during the ante and intra partum period to prevent transmission from mother to child (Connor et al., 1994). Subsequently, Drs. Marthas and Van Rompay developed a physiologically more relevant oral infection model of SIV in neonatal and infant rhesus macaques to study pediatric HIV pathogenesis and to test antiretroviral drug intervention strategies, and vaccines to prevent breastmilk transmission of HIV in human infants. The testing of 9-[2-(R)-(phophonomethoxy)propyl]adenine (PMPA or tenofovir) were instrumental in approving PMPA for human infant use and highlighted the need to adjust dosing, monitor toxicity for bone development and kidney function, and emergence of viral resistance mutants (Van Rompay et al., 1995; Van Rompay et al., 1998a; van Rompay et al., 1999; Van Rompay et al., 1999; Van Rompay et al., 2000). Longterm safety studies documented that even prolonged use of tenofovir in rhesus macaques from infancy to adulthood does not have teratogenic effects (Van Rompay et al., 2008) and that animals, even when they develop resistance mutants, can mount antiviral immune responses able to control virus replication after drug withdrawal (Van Rompay et al., 2012).

The oral SIV infection model aided in identifying viral dissemination from the site of virus exposure to systemic virus replication in lymphoid tissues and the brain, and how the infant immune system responded to HIV infection and impacted disease outcome, including impairment of central nervous system development (Abel et al., 2006; Hartigan-O’Connor et al., 2007; Curtis et al., 2014; Carryl et al., 2017; Amedee et al., 2018; Haddad et al., 2021). Over the years, the pediatric HIV model in rhesus and pigtail macaques was optimized by switching from a high-dose to a repeated lower dose oral viral exposure regimen to more accurately reflect HIV acquisition by breastfeeding in human infants, and by utilizing simian/human immunodeficiency viruses to improve the translational value of pathogenesis, host immunity, HIV vaccine (see below), and cure studies (Jayaraman et al., 2004; Van Rompay et al., 2006; Mavigner et al., 2018; Bricker et al., 2020; Nelson et al., 2020; Obregon-Perko et al., 2020; Bricker et al., 2022; Evangelous et al., 2023; Hora et al., 2023; Shapiro et al, 2024

Another important contribution of the pediatric NHP model was its role in pioneering passive antibody administration to protect against breast milk transmission of SIV/SHIV and in testing and optimizing HIV vaccine and cure strategies. Very early studies established that passive antibody administration improved disease outcome in SIV-infected infant rhesus macaques (Van Rompay et al., 1998b; Ferrantelli et al., 2004; Jaworski et al., 2013). These studies culminated in the testing of neutralizing antibodies and broadly-neutralizing antibodies as pre-and post-exposure prophylaxis in SHIV-infected newborn macaques (Hessell et al., 2015; Hessell et al., 2016; Hessell et al., 2018; Shapiro et al., 2020). Results from these studies undoubtedly informed human clinical trials with bNAbs (McFarland et al., 2021).

Initial HIV vaccine studies in infant rhesus macaques were aimed at protecting against breast milk transmission through an accelerated HIV vaccine regimen (Van Rompay et al., 2005b; Van Rompay et al., 2010; Marthas et al., 2011; Jensen et al., 2016b; Jensen et al., 2016a). With the introduction of bNAbs as potentially long-lasting means of protecting HIV exposed infants against HIV acquisition (see above), vaccine strategies were optimized for dose, interval and adjuvants (Phillips et al., 2017; Phillips et al., 2018; Singh et al., 2024) and current vaccine approaches are aimed at inducing bNAbs. These studies have repeatedly demonstrated that infant rhesus macaques can mount potent persistent binding antibody responses of high magnitude and that passive administration of a monoclonal bNAb does not interfere with vaccine-induced HIV Env-specific antibody induction (Dennis et al., 2019; Curtis et al., 2020). Most importantly, immunization with the germline targeting BG505 GT1.1 SOSIP trimer adjuvanted with the TLR7/8 agonist 3M-052 in stable emulsion resulted in the induction of CD4 binding site-targeting bNAb precursors in 4 of 5 infant rhesus macaques by week 150 (Nelson et al., 2024). The same SOSIP trimer has been evaluated in human adults (NCT04224701) with the TLR4-based adjuvant AS01B. Preliminary results suggest that the high dose BG505 GT1.1 SOSIP (330 mcg) regimen induced bNAb precursors in 72% of the vaccine recipients and follow-up trials are ongoing to determine whether a boost with the wt BG505 SOSIP trimer can further push the bNAb cell lineage. It is well accepted that the induction of bNAbs to protect against the majority of circulating strains of HIV worldwide will require a multidose vaccine regimen [reviewed in (Haynes et al., 2023)]. The findings obtained from infant rhesus macaque vaccine studies, in particular the recent the data from the BG505 GT1.1 immunization (Nelson et al., 2024), imply that the infant immune milieu is more favorable for the induction of bNAbs and provides the opportunity to exploit the global infant EPI immunization schedule for introduction of an HIV vaccine that can be boosted throughout childhood and provide protection against sexual HIV acquisition in adolescents. The pediatric NHP model, thus, represents an important resource in optimizing an effective HIV vaccine, by exploring different platforms, immunogens, adjuvants, and by defining an optimal dosing and interval for vaccine administration. Future studies should also include the impact of HIV Env bNAb cocktails on vaccine-induced antibody responses, and test for interaction between standard pediatric vaccines and the HIV vaccine.

## Ethical considerations and alternatives for research involving pediatric NHP models

Pediatric studies represent a relatively small proportion of NHP studies, and an even higher bar of ethical considerations is applied before approval of such studies is granted. The importance of awareness, recognition, and acknowledgment of special requirements for pediatric infection models by researchers cannot be over-emphasized. The early life period is not only a time of major development of the various organ systems, but also a time critical for behavior and social interactions throughout the lifetime. Questions to consider should include whether animals should be nursery-reared requiring almost immediate separation of the newborn from its mother, or if the nature of the study allows for dam-rearing of the infant NHP. Infants that are nursery-reared often present with behavioral problems that prevent re-introduction of these animals into the colony. If nursery housing is advised to avoid infection of the dam by contagious pathogens, co-housing of 2 or more infants is preferred. The NPRCs have developed enrichment programs specially tailored to young animals to improve the overall welfare of infant NHPs in biomedical research. Veterinary care of infant NHPs is more time consuming and requires special training. Therefore, infant NHP studies are extremely costly to perform, and studies involving viral pathogens requiring BSL-3 or higher containment are often cost-prohibitive. As a result, group sizes in pediatric NHP studies are generally smaller and, therefore, require thorough planning to achieve conclusive, biologically relevant, and statistically significant results.

**Figure 3:**
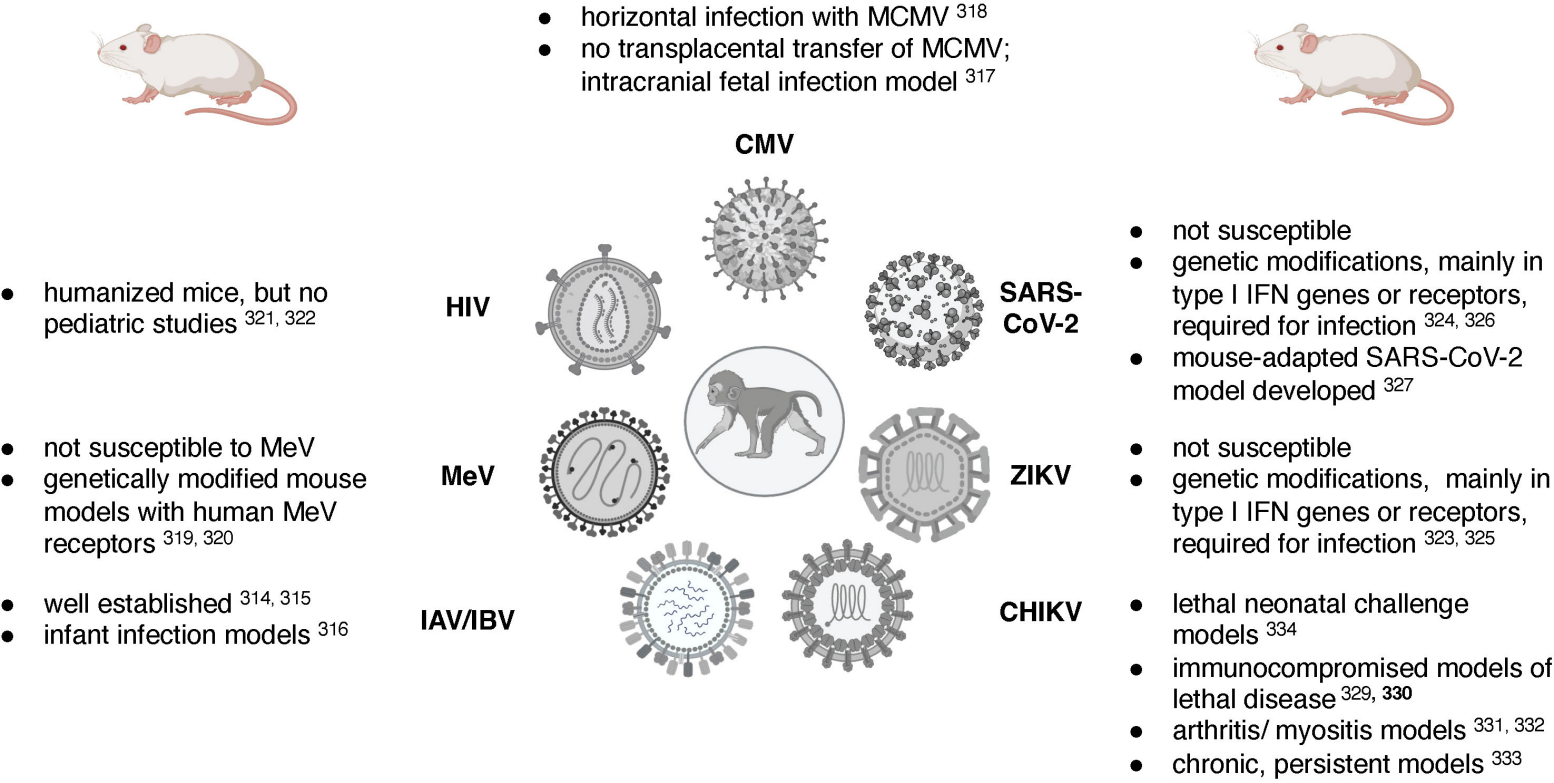
Challenges of mouse models for pediatric viral diseases. Figure created in BioRender.

Rodent, and in particular mouse models, have been established for many of the viruses discussed in this review. While it is beyond the scope of this review to compare and contrast pediatric mouse and NHP models, **Table 1** summarizes some of the key advantages and disadvantages of these two animal models and **Figure 3** lists some of the challenges in designing relevant mouse models of the specific viral infections reviewed here. The research tools available for the mouse model, including a wide-array of reagents such as antibodies, and the vast number of genetically modified mouse strains have positioned the mouse model as the model-of-choice to answer mechanistic questions. The mouse model of influenza virus infection presents a great example [see reviews by (Groves et al., 2018; Nguyen et al., 2021)], and pediatric infection models are available (Reuman et al., 1983). However, the biological differences in major developmental processes (e.g., immune ontogeny) and the species-specificity of some viral (and other) pathogens require animal models that are more closely related to humans, such as NHP. Murine CMV, for example, cannot be transmitted transplacentally and the study of congenital CMV requires direct intracranial infection of the fetus (Zhou et al., 2022) [see also review of MCVM by (Fisher and Lloyd, 2020)]. To study measles, mouse models genetically modified to express human measles virus receptors had to be developed (Rall et al., 1997; Ohno et al., 2007). While humanized mice are widely used for HIV pathogenesis, treatment and cure studies (Victor Garcia, 2016; Gillgrass et al., 2020), the type of human graft and the time required for human engraftment prohibit the use of humanized mice for pediatric diseases. Many of the newly emerging viruses, including ZIKV and SARS-CoV-2, cannot infect mice effectively, and genetic modification resulting in an immunocompromised host are needed to allow virus replication (Bradley and Nagamine, 2017; Knight et al., 2021). Nonetheless, relevant mouse of ZIKV infection have been established (Lazear et al., 2016; Bradley and Nagamine, 2017). In the case of SARS-CoV-2, one model was developed to account for genetic diversity in the population to better recapitulate the distinct pathogenesis outcomes in humans (Robertson et al., 2023), and another model adapted SARS-CoV-2 to the mouse host (Dinnon et al., 2020). These models, however, have not been applied to study infant SARS-CoV-2 infections. CHIKV can infect mice (Haese et al., 2016), and adult mouse models of lethal disease (Gardner et al., 2012; Rudd et al., 2012), arthritis (Gardner et al., 2010; Morrison et al., 2011), chronic and persistent disease (Hawman et al., 2013), as well as lethal neonatal challenge models (Couderc et al., 2008), have been developed.

**Table 1:** Advantages and disadvantages of mouse versus nonhuman primate models for human pediatric viral diseases.

Parameter	Mouse Model	Nonhuman Primate Model
Phylogenetic relationship to humans	more distant; rodents	close, primates
Anatomy and Physiology	somewhat similar^a^	highly similar
Immune System Development	important differences^b^	very similar
Infectious Housing Requirements	indoors (cages)	indoors (cages)
Handling and Care	relatively easy	specialized
Availability	high	somewhat limited
Costs	low to high	high
Genetic Variability	inbred^c^	outbred^d^
Specimen and Tissue Access	good	very good
Transgenic/ Knock-out/in Models	yes	no
Biological Reagents and Tools	widely available	more limited

a examples of differences include the female reproductive tract, lack of tonsils, different rates of senescence and metabolism; see also (Demetrius, 2005; Sieck, 2019; Ruberte et al., 2023).

b relevant for viral infections, there are important differences in type I interferon signaling pathways that impact viral responsiveness in mice compared to NHP; see also review by (Mestas and Hughes, 2004).

c genetic diversity can be addressed by using mice of the Collaborative Cross or outbred mice.

d genetic diversity is variable; Rhesus macaques in HIV research are predominantly of Indian origin; cynomolgus macaques in US NPRCs are often of Mauritian origin and present with limited genetic variability.

Tissue explant models and 3D tissue culture models present other alternatives to address specific questions of virus-host interactions (Hwang et al., 2023). Application and utilization of these novel tools is also encouraged by the FDA (Zushin et al., 2023), in particular for drug toxicity or adjuvant testing. Yet, adaptation of these models to pediatric diseases is slow, one exception is the development of *in vitro* systems to study human infant immune responses to vaccines and adjuvants (Diray-Arce et al., 2022; Morrocchi et al., 2024; Schuller et al., 2024).

## Summary of advantages and limitations of pediatric NHP models

Infant NHP models provide unique opportunities to better understand infant diseases and, therefore, to inform novel prevention and treatment strategies. An obvious advantage is sample access. Among the viral pathogens discussed in this review, CMV and Zika can impact the CNS, MeV, influenza and SARS-CoV-2 target the lung, HIV is a mucosal disease impacting the immune system throughout the body; pathogenic mechanism targeting these organ systems are difficult to decipher in human infants as it would often be unethical to collect samples from these tissues. Yet, virus-host interactions and determinants of pathogenesis are often dependent on the local milieu and specific cell-cell interactions at these sites. At the same time, sample size is limited in infants. Blood volumes cannot exceed 12 ml/kg in a 4-week period and the birth weight of, for example, a rhesus macaque is ranges from 350-600 g. Due to the size of the infant animal, biopsies (e.g., lymph nodes, bone marrow) are generally not performed prior to 3 months of age. Yet, the increasing availability of single-cell analysis methods allows for a comprehensive analysis of even small cell numbers (Smolen et al., 2014; Amenyogbe et al., 2015; Olin et al., 2018; Lee et al., 2019). In the case of chronic (e.g., long COVID), or even lifelong infections (e.g., HIV) or infection sequelae (e.g., from congenital CMV or ZIKV infection), the NHP model may allow for long-term longitudinal follow up studies that span the period from birth to adolescence (see **Figure 1**) in less than 4 years, whereas comparable studies in humans would take > 10 years. However, it needs to be determined how well the contracted age periods of NHP neonates, infants and juveniles reflect the progression of human babies to adolescents. This might be especially relevant in the testing of intervals for vaccines or the dosing of treatments. The genomes of several NHPs, including rhesus macaques, have been sequenced and allow for a better understanding of primate evolution (Kuderna et al., 2023). As these NHP genomes become fully annotated, they will allow to define the impact of host genetics on disease outcome, while also considering genetic differences between NHP and humans, in particular in immune responses genes.

## Conclusions and future opportunities

The goal of the review was to highlight how pediatric models of viral infections can contribute to our understanding of viral pathogenesis, but more importantly help inform novel prevention and intervention strategies in humans. Animal models are generally established to inform clinical diagnostics, treatment and prevention strategies in human adults. Yet, the distinct immune environment in infants and normal developmental changes in the endocrine and central nervous system prevent the simple extrapolation of “adult solutions” to the pediatric population. Treatment and vaccine regimens require dose and interval adjustments and potentially the inclusion of immune modulators (e.g., adjvuants) that optimize and enhance responses in infants. The contributions of the few viral infection models in infant NHPs presented in this review clearly highlight the values studies in pediatric models can have in informing treatment, such as the PMPA treatment in HIV-infected infants, or vaccine strategies, as discussed for influenza, SARS-CoV-2, and HIV. Clinical trials in the pediatric population could benefit from preclinical studies in relevant pediatric animal models that assess safety and determine optimal dosing of immunogens and adjuvants, and thereby reduce the risk of clinical trials and potentially the number of subjects required to obtain conclusive data.

The review is by no means comprehensive, a few select viruses were presented, and many aspects of the NHP models presented could not be discussed in great detail. Limited data were available to compare differences between NHP species infected with the same viral pathogen. Specific virus strain, dose, and route of viral exposure can greatly impact disease outcome to a viral pathogen, and this review only touched upon this importance (e.g., influenza, HIV viruses). Rarely are studies performed in parallel in different age groups to define age-specific pathogenesis and host immunity parameters to inform the need for potential age-specific intervention strategies.

Despite these drawbacks, pediatric NHP models provide unique opportunities, such as access to multiple tissues to study organ-specific effects of viral pathogens and paired dam-infants for the study of congenital infections, vertical transmission, and impact of passive maternal immune factors on infant immune responses. To be of high clinical relevance, and acknowledging that specific clinical question may require distinct animal models, pediatric NHP models would greatly benefit from more standardization, both in the model system and in the analysis of immune or other parameters (e.g., pharmacokinetics). The latter would also allow for more comparison and generalization of data. For example, data from different groups utilizing the same infection model could be combined to define virus-specific or host-specific factors impacting pathogenesis more conclusively, or immune responses to viruses targeting the same organ (e.g., lung for respiratory viruses; brain for ZIKA, HIV impact on CNS) could be compared between different viral models to increase our understanding of organ-specific responses in infants.

Our understanding of the pediatric immune system and its response to viral infection remains limited. Without doubt, insights obtained in pediatric NHP models into the pathogenies of fetal and early life viral infections, virus-host interactions, and immune responses would aid in limiting severe disease outcomes by informing diagnostics, intervention and treatment strategies, and developing efficacious vaccines tailored to the immune system of this vulnerable population.
